# Do blood plasma levels of oxytocin moderate the effect of nasally administered oxytocin on social orienting in high-functioning male adults with autism spectrum disorder?

**DOI:** 10.1007/s00213-016-4339-1

**Published:** 2016-06-02

**Authors:** Monika Althaus, Yvonne Groen, Albertus A.Wijers, Henriette Noltes, Oliver Tucha, Fred C. Sweep, Federica Calcagnoli, Pieter J. Hoekstra

**Affiliations:** Department of Child- and Adolescent Psychiatry, University of Groningen, University Medical Center Groningen, Hanzeplein 1, 9713 GZ Groningen, The Netherlands; Department of Clinical and Developmental Neuropsychology, University of Groningen, Grote Kruisstraat 2/1, 9712 TS Groningen, The Netherlands; Department of Experimental and Work Psychology, University of Groningen, Grote Kruisstraat 2/1, 9712 TS Groningen, The Netherlands; Lentis, Autism Team of the North of the Netherlands (ATN), Laan Corpus den Hoorn 102-2, 9728 JR Groningen, The Netherlands; Department of Laboratory Medicine, Radboud University Medical Center, Geert Grooteplein 8, 6525 GA Nijmegen, The Netherlands; Department of Behavioral Physiology, University of Groningen, Nijenborg 7, 9747 AG Groningen, The Netherlands

**Keywords:** Baseline plasma oxytocin, Post-oxytocin treatment plasma oxytocin, Social orienting, Autism spectrum disorder, Electrocortical and cardiac evoked responses

## Abstract

**Objective:**

The study investigated whether baseline plasma oxytocin (OXT) concentrations might moderate the effects of nasally administered OXT on social orienting.

**Methods:**

Thirty-one males with Autism spectrum disorder (ASD) and thirty healthy males participated in a double-blind placebo-controlled crossover trial. After administration of the compound, participants were viewing pictures from the International Affective Picture System that represented a systematic variation of pleasant, unpleasant and neutral scenes with and without humans. The outcome measures were a cardiac evoked response (ECR) and a cortical evoked long latency parietal positivity (LPP).

**Results:**

Males with ASD had significantly higher plasma baseline levels than the controls. In the absence of general treatment effects, higher *baseline* concentrations were found to be associated with larger treatment effects, particularly in the group of males with ASD. Higher *post-treatment* plasma OXT concentrations were found to be associated with smaller treatment effects and larger orienting responses in the placebo situation in the group of controls.

**Conclusions:**

We interpret our findings as suggesting that it is the central availability of OXT determining how much of the nasally administered OXT will become centrally absorbed and how much of it will become released into the bloodstream.

## Introduction

Following the many studies on the role of oxytocin (OXT) in prosocial behaviour and attachment (see Bartz et al. 2011; Guastella and MacLeod 2012; Bakermans-Kranenburg and van I Jzendoorn 2013 for extensive reviews), OXT has increasingly been investigated for its role in the pathophysiology and potential treatment of the core deficits in autism spectrum disorders (ASD), especially since its nasal administration has suggested some promising results (Andari et al. 2010; Guastella et al. 2010). ASDs are characterized by deficits in social communication and interaction as well as by restricted, repetitive behaviours and interests (American Psychiatric Association 2013).

In their review on ‘the role of oxytocin in psychiatric disorders’, Cochran et al. (2013) highlighted three lines of investigation on which studies into the social effects of OXT have focused: (1) studies of endogenous peripheral (blood plasma, urinary and salivary) and/or central (cerobrospinal fluid [CSF]) OXT levels, (2) genetic studies and (3) exogenous OXT administration studies. As the present study aimed at investigating whether baseline OXT plasma levels are associated with the effect of nasally administered OXT on social orienting in healthy male adults and male adults with ASD, we have briefly summarized the main findings of the OXT treatment studies conducted on people with ASD as well as the main findings of studies investigating endogenous peripheral and central levels of OXT in a variety of clinical and non-clinical groups.

The first randomized placebo-controlled studies on the acute effects of exogenously administered OXT on the behaviour of individuals with ASD were those by Hollander and colleagues (2003; 2007) who administered OXT by intravenous infusion to adults with autism and Asperger’s disorder. The experiments showed a greater reduction in repetitive behaviours during OXT infusion compared to placebo (2003) and suggested improvements in affective speech comprehension (2007). In a subsequent study, Guastella et al. (2010) administered OXT nasally to 12- to 19-year-old males with autism or Asperger’s disorder and demonstrated improved performance on the Reading the Mind in the-Eyes test (RMET, Baron-Cohen et al 2001) that measures the ability to infer the mood of a person from pictures where only the eye regions of faces are shown. Furthermore, increased cooperative interaction and self-reported feelings of trust were found after nasal administration in young adults with high-functioning autism and Asperger’s disorder when playing a computerized ball throwing game (Andari et al. 2010). These improvements in social behaviour refer to capacities of making inferences about another person’s mood or intention, i.e. to capacities that are related to having a theory of the other’s mind (ToM), the main ingredient for showing cognitive empathy (e.g. Shamai-Tsoory 2011). In the study by Andari and colleagues, moreover, increased fixation time was found on the eye region of faces in pictures that had to be judged for gender and gaze direction. Interestingly, this response to OXT depended on the patients’ social interaction style. Most of the patients who actively displayed approach behaviour, though in an inappropriate or one-sided way, showed positive effects, while most of the individuals who avoided or actively rejected proximity with others showed no response to the treatment. This indicated that the response to OXT may differ for subgroups of patients with ASD. Two longer-term trials have been conducted administering OXT nasally twice daily for 6 weeks (Anagnostou et al. 2012) and once daily for four consecutive days (Dadds et al. 2014) to adults and children with ASD, respectively. Both studies found no significant change in the primary outcome measures of general social functioning and repetitive behaviours, i.e. the core features of ASD. Although the first study found improvements in emotion recognition as measured with the RMET as well as in a broad measure of quality of life, there were no such findings in the second study. The main conclusion by Dadds and colleagues was therefore to be cautious in recommending nasal OXT as a general treatment for young people with autism.

This suggestion was corroborated by the findings of our own study on the effects of nasally administered OXT on social orienting in male adults with ASD (Althaus et al. 2015). In the first place, this study revealed no significant treatment effects, neither in the group of healthy control males nor in the group of males with ASD. However, moderator analysis demonstrated that OXT did enhance social orienting in male adults with ASD who are easily distressed when seeing others in stressful situations, and in healthy males who are highly sensitive to anticipated punishment and criticism or have a low drive for goal achievement. We concluded that individual differences in stress-related avoidance tendencies should be taken into account when considering OXT as a treatment of social deficiencies in autism, which was in agreement with what Dadds et al. (2014), Bartz et al. (2011), and Andari et al. (2010) had previously pointed to, i.e. the person and context dependency that might explain the inconsistencies found in reported OXT effects.

Another moderator of OXT administration effects not yet investigated as such might be the natural variation in endogenous OXT levels. These natural variations are most often measured in a peripheral medium such as blood plasma, urine or saliva, given the discomfort and risks associated with taking CSF by lumbar puncture. Hence, studies of CSF-OXT in humans are scarce and therefore still little is known about the relationship between the central and peripheral availability and action of OXT.

Increased plasma and saliva levels of OXT have repeatedly been found to be related to prosocial behaviour and hence have been suggested to reflect central OXT effects. For example, peripheral OXT levels were elevated in mothers after interacting with their infants (Strathearn et al. 2009). Also, higher plasma OXT levels during pregnancy in primaparous women predicted higher quality of postpartum maternal bonding behaviour (Feldman et al. 2007), while lower levels were predictive of postnatal depression (Skrundz et al. 2011). In the same line, lowered CSF-OXT levels were found in adult women who had been exposed to childhood maltreatment (Heim et al. 2009), and lowered urinary OXT levels were found in children placed in orphanages shortly after birth (Fries et al. 2005). Also in patients with schizophrenia, plasma OXT levels were found to be lower than in healthy subjects, while, in addition, lower levels were associated with more psychotic symptoms (Rubin et al. 2010).

However, the relationship between peripheral OXT levels and social function in ASD is not as straightforward as the above studies would suggest. That is, while lower mean plasma OXT levels were found in 6- to 11-year-old boys with autism compared to age matched healthy controls, elevated OXT levels were associated with lower scores on the Vineland Adaptive Behaviour Scale (VABS), which was reverse in the control group (Modahl et al. 1998; Green et al. 2001). This suggests that (high) OXT plasma levels are differentially related to centrally controlled behaviour in autistic vs. healthy children. Yet, in contrast to what has been found for children with ASD, in a study comparing adults with ASD to healthy control adults, baseline blood plasma levels were shown to be significantly higher in the ASD group. In this study, no correlations between plasma OXT levels and impairments in social interactive behaviour were found (Jansen et al 2006). Different from the studies with children, however, the adults were all high functioning while intellectual functioning in the studies of children varied among the participants. This suggests that the OXT system of individuals with ASD may change during lifespan and that OXT plasma levels in ASD may not only be related to developmental but also to intellectual determinants (Jansen et al. 2006).

In summary, the majority of studies suggest that high plasma OXT baseline levels are associated with prosocial skills and behaviour. With regard to the moderating effects of plasma OXT baseline levels on the effect of exogenously administered OXT on social behaviour, this might mean that individuals with lower baseline levels would benefit more from OXT administration and hence show larger treatment effects than those with higher baseline levels.

The present study used the data of our abovementioned previous study showing that nasally administered OXT enhances social orienting in a selective group of male adults with ASD (Althaus et al. 2015). While our previous study took into account the OXT effect-moderating influences of personal characteristics as assessed by questionnaires, the present study investigated whether the baseline level of blood plasma OXT influences the effect of nasally administered OXT on social orienting. As in our previous study, social orienting was investigated by comparing neurophysiological responses to affective pictures with and without social relevance. These responses were an evoked cardiac response (ECR, i.e. stimulus-dependent heart rate slowing) and an event-related potential (ERP) derived from the electroencephalogram (EEG), i.e. a late long lasting parietal positivity (LPP). Both types of responses, in particular to the pictures with humans, have previously been shown to discriminate between men and women (Proverbio et al. 2009; Groen et al. 2013; Althaus et al. 2014) and to correlate with empathy-related questionnaire measures (Groen et al. 2013; Althaus et al. 2014).

In agreement with the study by Jansen et al. (2006), we expected that higher blood plasma OXT baseline levels would be found in the group of male adults with ASD than in the group of male controls. Furthermore, we explored whether blood plasma OXT levels correlated with behaviour characteristics as assessed by means of a variety of questionnaires. We finally expected that males with lower blood plasma OXT baseline levels would benefit more from exogenous OXT administration and hence would show larger treatment effects. This would be reflected by an enhancement of the ECR and LPP responses to affective pictures with humans as compared to pictures without humans after OXT intake. As OXT plasma levels might be differentially related to centrally controlled behaviour in autistic and healthy people (see Modahl et al. 1998; Green et al. 2001; and Jansen et al. 2006), we explored whether the potentially moderating effects might differ for the male adults with ASD and the healthy male adults by investigating these effects in both groups separately.

## Methods and material

The study had been approved by the Medical Ethics Committee of the University Medical Center Groningen, and written informed consent was obtained from all participants.

Most parts of the methods and material have been described in more detail previously (Althaus et al. 2015). Yet, the parts most essential to the present paper have been outlined again below, completed by the description of OXT extraction and analysis.

### Participants

In total, there were *n* = 61 participants in the age of 18–34 years (M = 22.67; SD = 4.22). The patient group consisted of *n* = 31 males with a Diagnostic and Statistical Manual of Mental Disorders (DSM)-IV diagnosis of ASD, and the control group included *n* = 30 healthy male participants. The healthy participants were recruited by advertisements at schools and public spaces. They all reported to be free from psychopathology, which was confirmed by normal scores on the 90-item Symptom Checklist (SCL-90, Arrindell and Ettema 1986). The patients were recruited via our own outpatient clinic of the University Center Child and Adolescent Psychiatry, Accare, and via the Autism Team of the North of the Netherlands (ATN). The inclusion criterion for the ASD patient group was the presence of a DSM-IV-TR diagnosis in the autistic spectrum, i.e. autistic disorder, Asperger’s disorder or pervasive developmental disorder not otherwise specified (PDD-NOS, American Psychiatric Association 2004). For confirmation of the diagnosis established by a well-trained psychiatrist, the patient needed to have a score at or above the cutoff criterion for ASD on at least one of four standardized measures: the Communication or Social Interaction scale of the Autism Diagnostic Observation Scale (ADOS, Lord et al. 2000), which had been assessed in part of the sample as part of routine clinical care; the Social Responsiveness Scale for Adults (SRS-A, Constantino et al. 2003), which was completed by a good acquaintance of the participant; or the autism questionnaire (AQ, Baron-Cohen and Wheelright 2001) completed by the participant himself. Further inclusion criteria applying to all participants were IQ ≥ 80 as measured by the Groningen Intelligence Test (GIT-2, Luteijn and Barelds 2005); normal or corrected to normal vision, and being free from a nasal congestion due to a cold or allergy at the day of testing.

Exclusion criteria for all participants were regular use of psychotropic medication or having a history of alcohol or drug abuse. All participants had to abstain from taking alcohol, stimulants, ecstasy or soft drugs for 20 h, from caffeine for 4 h and nicotine for 2 h before the experiment.

The groups did not differ in age and intelligence. Furthermore, participants completed a set of questionnaires on autistic traits, empathic and social skills that were used for correlational analyses (see section 2.5.3).

### Procedure

Participants were invited for the experiment on two different days, separated by 1 week. During the experiment, participants were asked to view a series of photographs (see section 2.3) while their EEG and ECG were recorded. On one of the 2 days, participants received a nasal spray with OXT, 24 IU Syntocinon® (Sigma Tau), while on the other day, the nasal spray contained placebo, a saline solution (8 mg/ml; Pharmachemie, Haarlem, The Netherlands). The order of the treatments was double-blind and randomized within the groups. The experiment always took part at the same time of the day; either participants started at about 9 am (*n* = 15 controls and *n* = 11 males with ASD) or at about 2 pm (*n* = 14 controls and *n* = 20 males with ASD).

**Fig. 1:**
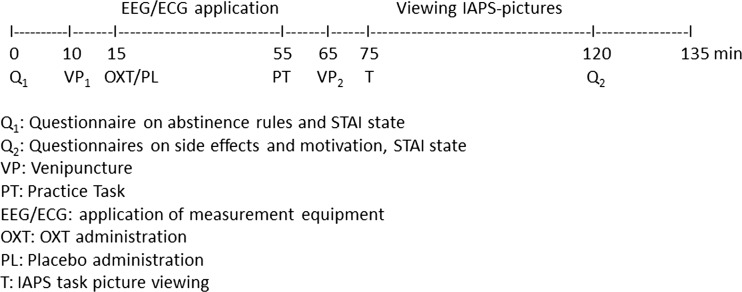
Time schedule of the experiment

The procedure followed the time schedule depicted in Fig. 1. Participants started with a check on the rules of abstinence and a brief questionnaire on state anxiety (Spielberger’s State-Trait Anxiety Inventory, STAI; Spielberger 1973). This was followed by a first venipuncture, with the tube being set on ice and immediately brought to the laboratory. Then the nasal spray was administered. After the experimenter primed the spray, the participant self-administered the spray by alternatingly puffing three times in each nostril (with 4 IU Syntocinon per puff this resulted in 24 IU in total). Participants were instructed to close one nostril and to ‘sniff-like’ inhale the puff in the other nostril. After spraying, the EEG and ECG equipment was applied by the experimenter (30 min), and a practice block of viewing pictures was performed by the participant (5 min). This was followed by a second venipuncture, with the period between spraying and the venipuncture lasting about 50 min (M = 53.13 min; SD = 4.92). The viewing of pictures started about 10 min later, and lasted for 45 min. The period between spraying and starting the task lasted about 60 min (M = 60.33; SD = 5.48). After task completion, the EEG/ECG equipment was taken off, and the participants completed some brief questionnaires about potential side effects of the nasal spray, their experience and motivation during the task, as well as their state of comfort/anxiety (STAI).

### IAPS Task

Participants watched a series of 414 photographs that had been selected from the International Affective Picture System (IAPS) (Lang et al. 2008), representing the following conditions: neutral, positive or negative emotions in scenes with humans (socially relevant conditions); or neutral, positive or negative emotions in scenes without humans (socially irrelevant conditions, see Groen et al. (2013) or Althaus et al. (2014, 2015)). By using E-prime 2.0, the pictures were serially presented for 1 s with a variable inter-stimulus interval of 3 to 5 s and required no response. The conditions were balanced and presented in random order. To ensure attentive watching, participants were instructed to press a button whenever a red-white checkerboard appeared on the screen. These target stimuli appeared randomly in a target/picture ratio of 1:6. Picture presentation was divided into three blocks of each lasting about 13.5 min, separated by short breaks of a few minutes.

### Data collection, preprocessing and dependent variables

#### Blood sampling and plasma OXT analysis

Blood samples were obtained using 10 ml EDTA tubes that were immediately put on ice and centrifuged at 1500 g for 15 min within 1 hour after collection. To separate OXT from its binding proteins, 300 μl HCl (0.5 M) was added to 1 ml of EDTA plasma, and the mixture was prepurified by means of Oasis HLB (1 ml, 30 mg) extraction cartridges. OXT was eluted from the cartridges by 100 % methanol. The eluates were evaporated in a Savant SpeedVac SC200, and 250 μl phosphate buffer (Na_2_HPO_4_·_2_H_2_O containing 13 mM EDTA, 0.02 % sodiumazide, 0.25 % BSA, 0.1 % Triton X-100, pH 7.4) was added to the tubes. OXT was quantified by an in-house radioimmunoassay (RIA). In short, 50 μl polyclonal antibody (diluted 1:160,000) raised in rabbit was added to 100 μl reconstituted sample and pre-incubated at 4 °C for 72 h. Then, 25 μl of ^125^I-labelled OXT (3000 dpm) was added, and incubation was continued for 24 h. Bound and free OXT were separated by a second antibody (10 % sheep anti-rabbit IgG and 0.01 % rabbit IgG (carrier protein)/polyethyleneglycol 6000 solution. Radioactivity in the tubes (bound fraction) was counted using an automatic gamma-counter (1470 Wizard TM Wallac, Turku, Finland). Data were analysed with a four parameter fit program.

Recovery was determined by addition of synthetic OXT (Sigma-Aldrich, cat. Nr O-6379) to plasma pools with 1.4 and 2.9 pmol/l endogenous OXT. The average recovery at these two levels (*n* = 5) was 78 ± 6 %. Within- and between-assay CVs were 11.1 and 12.8 % at 6.3 pmol/l. All samples were measured in duplo. The synthetic OXT preparation was used for radioiodination and for constructing a dose-response curve. The RIA specifically detects OXT. Cross-reactivity with arginine vasopressin, lysine-vasopressin and desamino-D-arginine-vasopressin all were <0.01 % on mass basis. The analytical sensitivity was 1.5 pmol/l. For statistical analysis of the data, levels below the analytical sensitivity of the assay were assigned a value of 50 % of this sensitivity.

#### ECG recording and pre-processing of the ECR

The participants’ ECG was recorded from pre-cordial leads by BrainRecorder (brain products) with a sample rate of 500 Hz. For analysis of the ECRs, sequential interbeat intervals (IBIs) occurring around each stimulus presentation were extracted from the R-peak series. IBIs were extracted in segments of 1 s before stimulus onset to 4.5 s after stimulus onset, and sampled every 0.5 s. This resulted in 12 IBI values for each stimulus. For each participant, averages were computed for each stimulus condition, with IBI0 referring to the IBI at stimulus onset and with IBI-1 serving as the baseline value (i.e. the IBI value at 1 s before stimulus onset was subtracted from all successive IBI values). Finally, by using SPSS, for each participant, the maximal cardiac deceleration was determined in the interval from −0.5 to 4 s (IBI_MAX_). For the validity of this measure, we refer to Althaus et al. (2014).

#### EEG recording and pre-processing of the LPP

EEG was also recorded by BrainRecorder from 61 scalp electrodes that were placed according to the 10–20 system by means of a lycra stretch cap (Electro-Cap Center BV). Based on previous work, 34 electrode positions were selected for analysis.

**Fig. 2:**
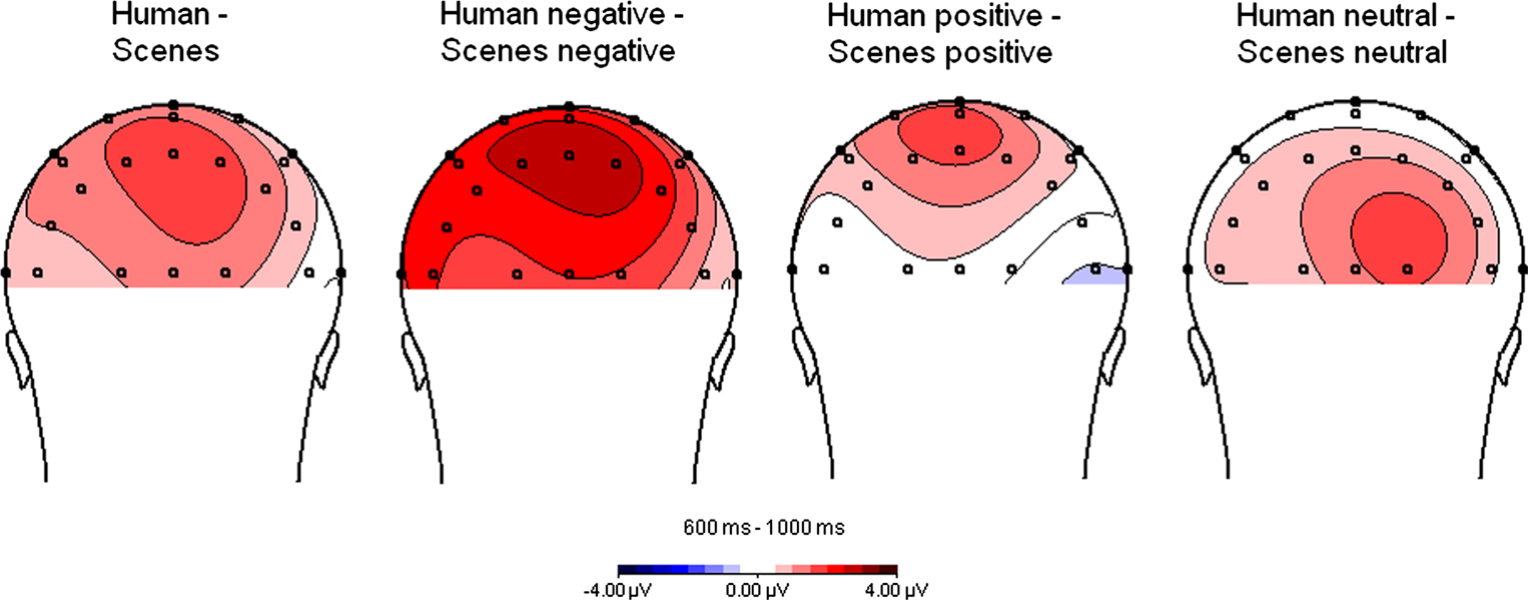
Topographical maps of the difference waves between pictures with and without humans in the interval 600–1000 ms after stimulus onset. Maps refer to the control group in the placebo condition and show the activity evoked by watching pictures with humans as compared to pictures without humans

In the placebo condition, the LPP amplitude was maximal over parietal electrode positions in the time window of 400–1000 ms after stimulus onset, see Fig. 2. To determine the electrode positions showing the strongest task effects, we performed repeated measures analyses on the data from the control group with the factors ‘hemisphere’, emotional ‘valence’ and presence of ‘humans’ on successive time intervals of 100 ms. We found that the largest effects of humans, valence and their interaction were present at Pz, P3 and P7 in the intervals running from 600 to 1000 ms. Further analyses of the LPP were therefore performed for the Pz, P3 and P7 potentials occurring in the interval running from 600 to 1000 ms.

### Statistical analyses

#### Group differences in OXT baseline and OXT post-administration levels

As the two blood plasma values obtained before OXT and placebo administration, respectively, did not differ from each other, baseline OXT (BLOXT) levels were computed by averaging these two values. This resulted in a more stable measure with less variance. Post-administration levels were adjusted for individual differences in pre-administration levels by subtracting the pre-administration values from the post-administration values (ΔOXT). All values were tested for group differences by means of Student’s *t* tests for independent samples. Differences between pre- and post-administration values were tested by paired Student’s *t* tests.

To check whether the baseline levels (BLOXT) measured in the morning would differ from those that were measured in the afternoon and whether this would differ for the two groups of participants, an ANOVA was carried out with the between subjects (BS) factors ‘group’ (controls vs. ASD) and ‘Time of the Day’ (morning vs. afternoon).

#### Correlations between peripheral OXT levels and behavioural measures

Pearson’s correlations were computed for both BLOXT and ΔOXT with all of our behaviour measures including age, intelligence and the questionnaire scores, which are summarized in Table 1.

**Table 1:** Relevant behaviour characteristics as measured by questionnaires

Questionnaire	Authors and year	Scales	Measurement
Autism spectrum quotient (AQ)	Baron-Cohen et al. 2001	AQ total	Autistic traits
Social Responsiveness Scale (SRS)	Constantino et al. 2003	Total Social awareness Social communication Social motivation Rigidity and repetition	Autistic traits
Empathy quotient (EQ)	Baron-Cohen and Wheelwright 2004	EQ total	Empathic skills
Interpersonal Reactivity Index (IRI)	Davis 1983	Personal distress Emotional concern Perspective taking Fantasizing	Empathic skills/personal distress
BIS/BAS questionnaire	Carver and White 1994; Gray and McNaughton 2000	BIS BAS drive BAS fun BAS reward	Avoidance and approach behaviour
Spielberger’s state-trait anxiety questionnaire (STAI)	Spielberger 1973	State anxiety Trait anxiety	State and trait anxiety

#### Treatment moderating effects of OXT baseline level: analyses of co-variance

Treatment effects on the ERC (IBI_MAX_) and the three LPP values referring to respectively, Pz, P3 and P7 were analysed by a 2*2*3 repeated measure ANOVA with the within subject (WS) variables treatment (placebo vs. OXT), human (humans vs. scenes), and valence (neutrals vs. positive vs. negative). To investigate the treatment moderating effect of plasma OXT, the variable BLOXT was entered as a covariate while focussing on the interaction of the covariate with the treatment (by task) effects. Analyses of covariance were carried out both on the whole group and the two groups separately. For effects with valence involved, Greenhouse-Geisser adjusted *p* values, and the epsilon correction factors were reported. Planned comparisons were carried out by computing contrasts between each of the three valence pairs. All data were controlled for outliers being defined as z ≤ −3 and z ≥ 3.

For all comparisons, next to the *p* values, Cohen’s *d* or partial eta squared are presented as measures of effect size. Rejection level was set at *p* = .05, but trend significant (*p* ≤ .1) findings were further elaborated if accompanied by medium or high effect sizes.

#### Regression analyses and high vs. low plasma OXT group comparisons

To investigate in which direction OXT effects on the ECR and LPP responses might have been moderated by plasma OXT, we conducted regression analyses of the BLOXT values on the treatment-dependent effect scores. To this end, the effects of (1) human and (2) human*valence were translated into the effect scores (delta values). For their exact computation, we refer to our previous study (Althaus et al. 2015). In a final step (trend), significant regressions, i.e. (trend) significant treatment interactions with the covariate were further elaborated by comparing groups with high vs. low BLOXT values on their orienting responses in both the placebo and OXT condition. This comparison was conducted by a 2*2*3*2 ANOVA design with the WS variables; treatment, human and valence and the BS variable group formed on the basis of the 33.3th and 66.6th percentiles of the BLOXT values.

## Results

### Task effects, OXT treatment effects and group differences in task and treatment effects

Both the ECR and LPP were larger in response to pictures with humans and this held for in particular the affective pictures. Yet, our study revealed no group difference and no effects of the nasally administered OXT on the orienting responses. Nor did the groups differ in their treatment response. For the statistical details and corresponding figures presenting the ECR and LPP in placebo and OXT condition, we refer to Althaus et al. (2015).

### Group differences in BLOXT levels and correlations of BLOXT levels with behaviour variables

First of all, morning values of BLOXT did not differ from afternoon values [*F*(1,56) = 0.16; *p* = .69], and this did not differ for the two study groups [*F*(1,56) = 0.25; *p* = .61].

Table 2 summarizes group means and standard deviations of the blood plasma OXT values obtained from the blood samples taken before and after placebo and OXT administration of the control group and the group with ASD, respectively. It shows that the groups differed significantly in their values of the pre-treatment (placebo and OXT) situations with small effect sizes, and in the values averaged across these situations (BLOXT) with moderate effect size. The males with ASD showed significantly higher baseline OXT plasma concentrations than did the control males.

**Table 2:** Group means and differences in plasma OXT values

	Controls *n* = 30	ASD *n* = 31	Group differences		
	Mean (sd)	Mean (sd)	t (df)	*p*	*d*
OXT^a^ before PL^b^	0.66 (0.93)	1.50 (2.07)	−1.96 (59)	.05	*0.39*
OXT after PL^b^	0.80 (1.33)	0.83 (0.95)	−0.89 (59)	>.1	0.23
OXT before OXT^c^	0.67 (0.94)	1.32 (1.67)	−2.37 (59)	.02	*0.47*
OXT after OXT^c^	9.88 (6.34)	9.60 (5.04)	0.19 (57)^d^	>.1	0.05
BLOXT	0.67 (0.77)	1.34 (1.05)	−2.85 (59)	.006	*0.73*
ΔOXT	9.20 (6.10)	8.22 (5.11)	0.67 (57)^d^	>.1	0.17

^a^Plasma OXT measured in pmol/l

^b, c^Placebo/OXT administration

^d^There were two missing values in the ASD group

BLOXT: mean value of OXT concentrations before placebo and oxytocin administration. ΔOXT: OXT after OXT^c^ minus OXT before OXT^c^

Large differences were found between the OXT levels before and after OXT administration [for the whole group: *t* (59) = 11.95; *p* < .001; *d* = 1.55; controls: *t* (29) = 8.26; *p* < .001; *d* = 1.51; ASD: *t* (28) = 8.67; *p* < .001; *d* = 1.61], yet groups did not differ in these ΔOXT levels [*F*_Time*Group_ (1,57) = .45; *p* = .51].

BLOXT did not correlate with any of the behaviour measures. It did, however, correlate significantly with the level of intelligence in the whole group (*r* = .31; *n* = 61; *p* = .02) and in the group with ASD (*r* = .38; *n* = 31; *p* = .04).

### Baseline OXT level as moderator of the OXT treatment effect

We found a significant interaction of BLOXT with the OXT treatment effect on responses to pictures with humans (as compared to the pictures without humans) for the ECR [*F*_Treatment*Human*BLOXT_ (1,27) = 5.69; *p* = .024; *η*^*2*^ = .17] and a trend significant interaction with medium effect size for the LPP at Pz [*F*_Treatment*Human*BLOXT_ (1,29) = 3.35; *p* = .07; *η*^*2*^ = .10] in the group with ASD, but not in the control group. However, the two groups did not differ significantly from each other in this BLOXT-dependent response to the treatment [ECR: *F*(1,27) = 1.22; *p* = .27; *η*^*2*^ = .02; LPP at Pz: *F*(1,59) = 2.53; *p* = .12; *η*^*2*^ = .04]. For the LPP at P7, a significant interaction was found for the whole group of participants [*F*_Treatment*Human*BLOXT_ (1,59) = 3.96; *p* = .04; *η*^*2*^ = .06].

**Fig. 3:**
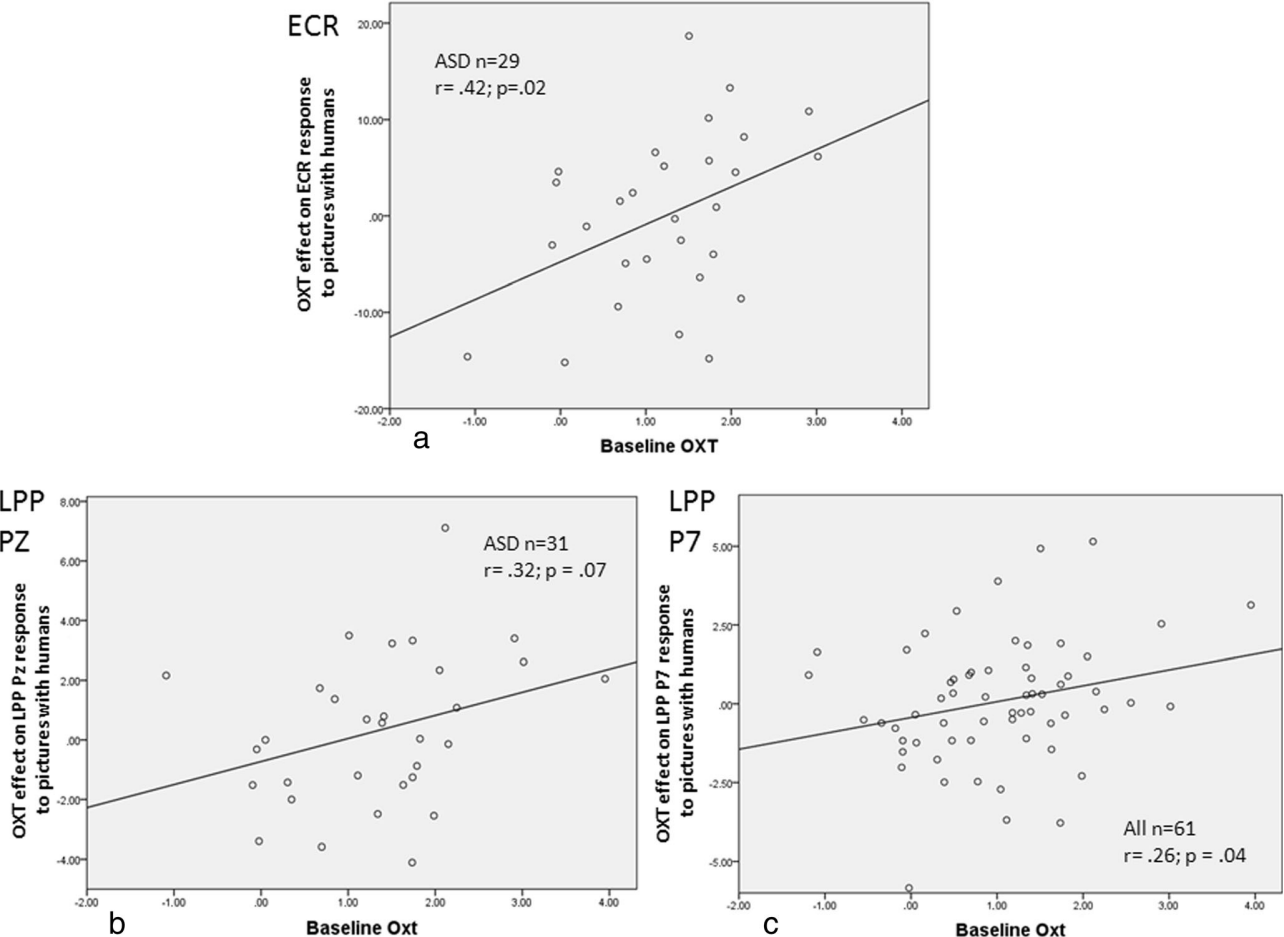
Regression of the baseline OXT concentrations upon the treatment effect on the orienting responses ECR (**a**), LPP at Pz (**b**) and LPP at P7 (**c**) to pictures with humans as compared to pictures without humans in male adults with ASD

Regression of the BLOXT values upon the OXT effect on the ECR response to pictures with humans shows that in the group with ASD lower BLOXT values were associated with smaller effects. The same holds for the regression of BLOXT upon the OXT treatment effect on the LPP responses to human pictures (Fig. 3a–c). For none of the orienting responses, BLOXT-moderating effects were found in the placebo situation.

**Fig. 4:**
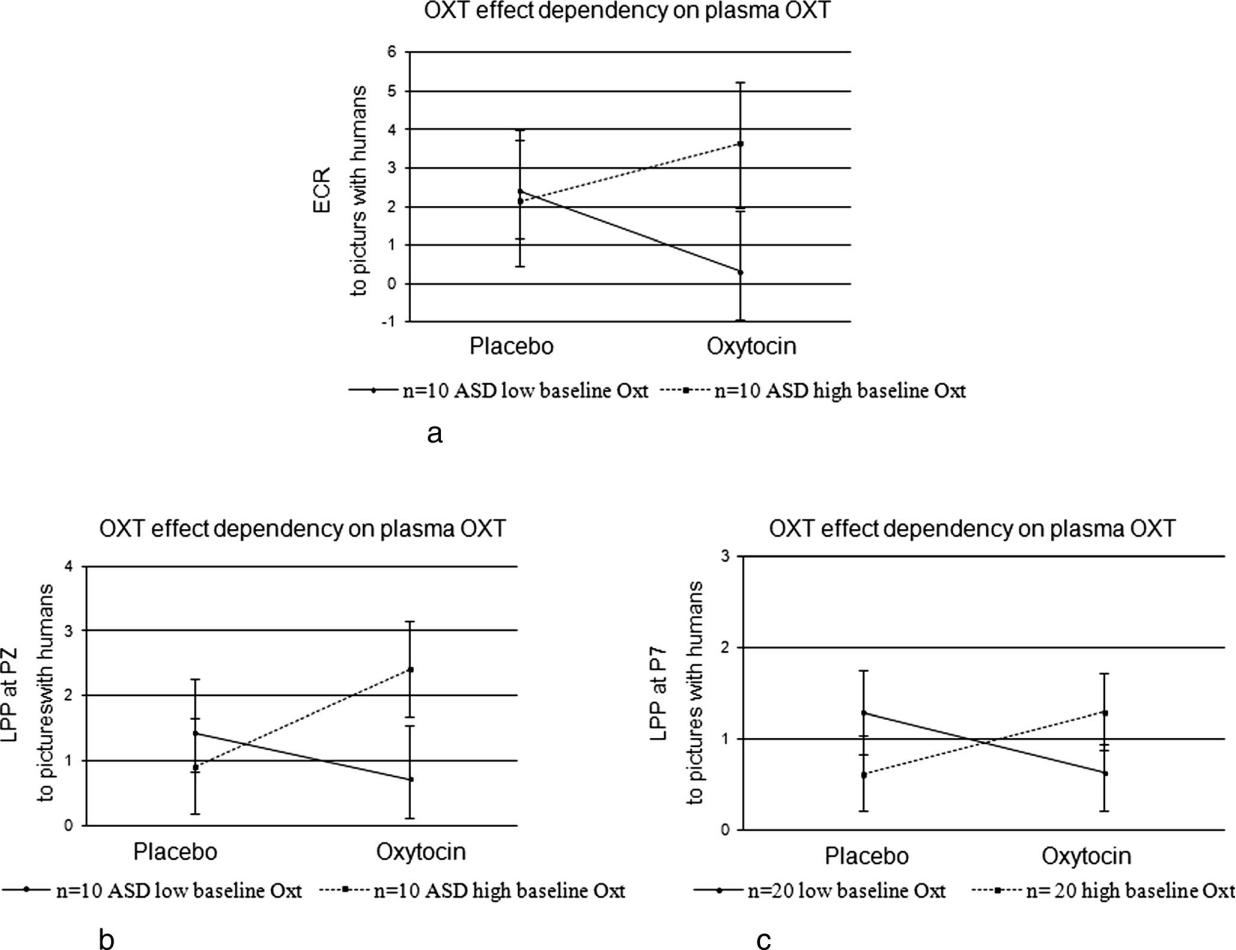
Means and SEMs of the human effect illustrating the increase of the orienting responses ECR (**a**), LPP at Pz (**b**) and LPP at P7 (**c**) to pictures with humans in the OXT condition for only the ASD males with high baseline levels of blood plasma OXT

Comparisons of the 33.3 % males (*n* = 10) with ASD showing the lowest BLOXT levels (≤0.90 pmol/l) with the 33.3 % (*n* = 10) males showing the highest BLOXT levels (≥1.74 pmol/l) resulted in a trend significant interaction of this group variable with the treatment effect on the ECR response to human pictures with high effect size [*F*_Treatment*Human*BLOXTGroup_ (1,18) = 2.99; *p* = .1; *η*^*2*^ = .15]. For the LPP at Pz, comparison of the two groups revealed a significant treatment by group interaction [*F*_Treatment*Human*BLOXTGroup_ (1,18) = 4.30; *p* = .05; *η*^*2*^ = .19], and for the LPP at P7, when comparing the 33.3 % (*n* = 20) participants of the whole group showing the lowest BLOXT values (≤0.49 pmol/l) with the *n* = 20 participants showing the highest BLOXT values (≥1.38 pmol/l), a significant group interaction with the treatment effect was found [*F*_Treatment*Human*BLOXTGroup_ (1,38) = 6.12; *p* =. 02; *η*^*2*^ = .14]. The interactions are plotted in Fig. 4a–c.

**Fig. 5:**
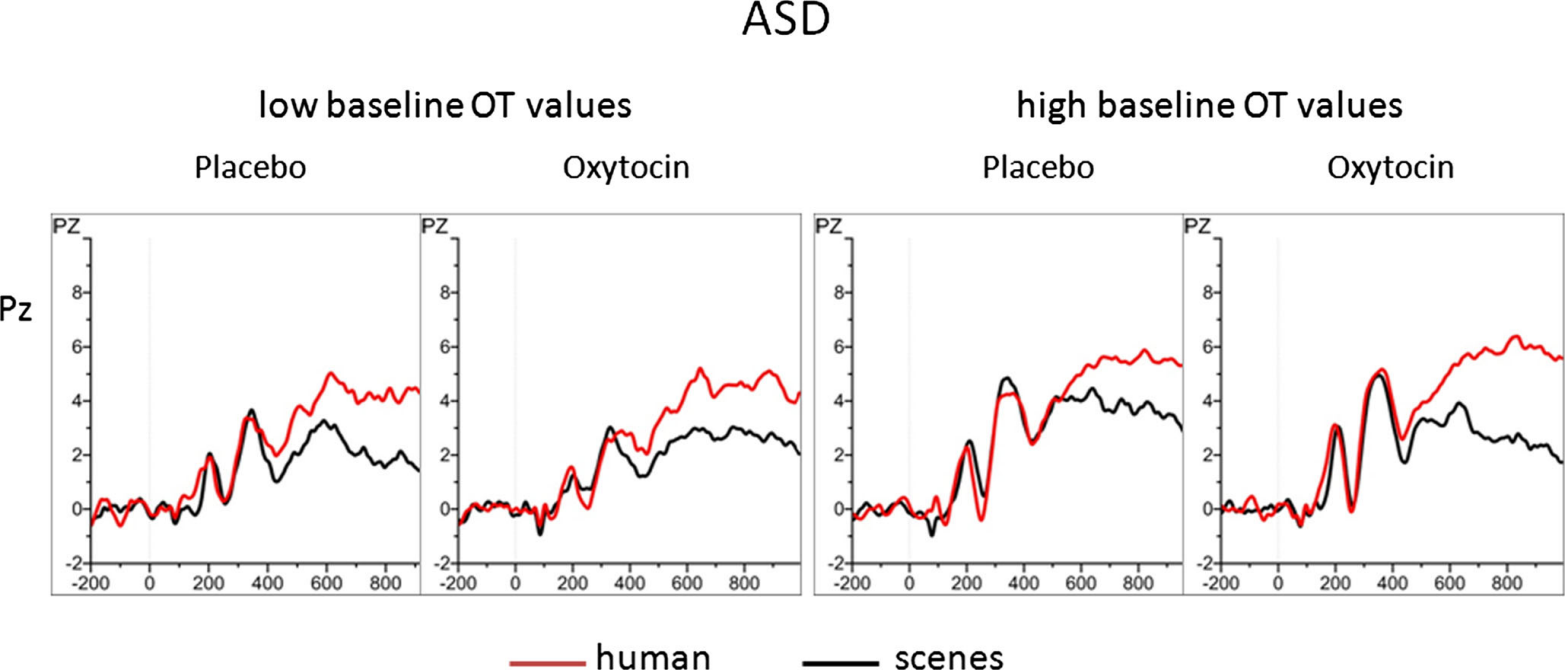
ERPs (LPP at Pz) in response to pictures with and without humans of ASD males having low (≤P33) and high (≥P66) blood plasma OXT baseline values

Summarizing the moderating influence of baseline OXT on the OXT treatment effects, we found that, opposite to what was expected, males with higher plasma OXT concentrations showed larger OXT treatment effects as expressed by an enhancement of their ECR and LPP responses to pictures with humans during OXT intake as compared to the placebo situation. This was found in particular for the males with ASD. An illustration of this OXT administration-induced enhanced orienting to pictures with humans in only the high baseline OXT group is given in Fig. 5 showing the LPP response at Pz, which discriminated the two groups of ASD participants with, respectively, low and high BLOXT values from each other with high effect size.

### Additional analyses

To obtain more insight into the mechanisms of how peripheral OXT levels may influence OXT administration- induced central effects, we investigated whether plasma OXT levels assessed *after* nasal OXT administration would be related to the effects of nasal OXT administration on social orienting. If larger peripheral post-administration levels were found to be associated with greater OXT treatment effects, this would suggests a parallel increase of peripheral and central levels after nasal OXT administration. If, however, larger post-administration plasma levels were associated with smaller treatment effects, this might suggests that less OXT had become centrally active due to, for example, leakage, less absorption or central saturation. Analyses were carried out on the baseline-adjusted post-administration levels (ΔOXT).

**Fig. 6:**
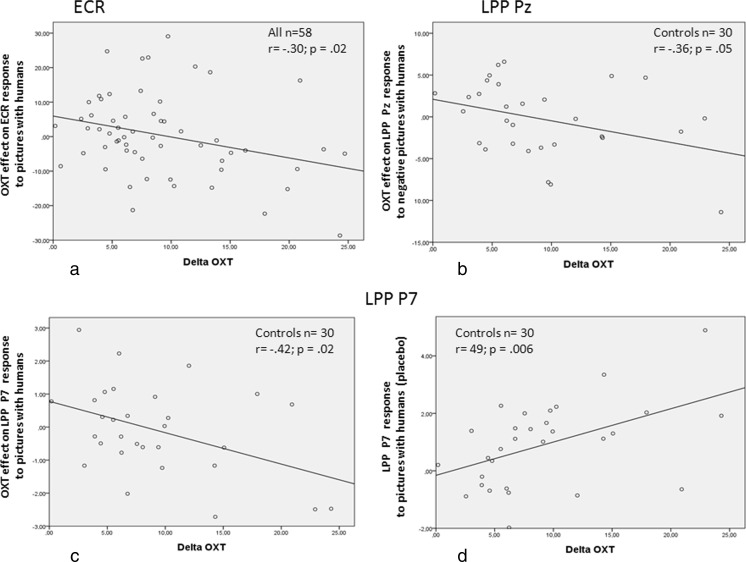
Regression of the baseline-adjusted post-administration OXT concentrations upon the treatment effects on the orienting responses ECR (**a**), LPP at Pz (**b**), LPP at P7 (**c**) to (negative, Pz) pictures with humans and upon the LPP P7 orienting responses in the placebo situation (**d**)

Regression of the post-administration values upon the OXT effect on the ECR responses to pictures with humans showed that in the whole group of participants lower ΔOXT values were associated with larger effects (*c* = 6.21, *ß* = −.30; *n* = 61; *t* = −2.34; *p* = .02). The same was found for the regression of the OXT treatment effect on the LPP Pz response to negative human pictures, yet in only the control group (*c* = 2.10, *ß* = −.36; *n* = 30; *t* = −2.02; *p* = .05) and the regression on the LPP P7 response to human pictures (*c* = 0.78, *ß* = −.43; *n* = 30; *t* = −2.49; *p* = .02) again in only the control group (see Fig. 6a–c). Moreover, a significant positive association was found between ΔOXT and the LPP P7 response to pictures with humans in the placebo situation (*c* = - 0.16, *ß* = .48; *n* = 30; *t* = 2.95; *p* = .006; see Fig. 6d).

**Fig. 7:**
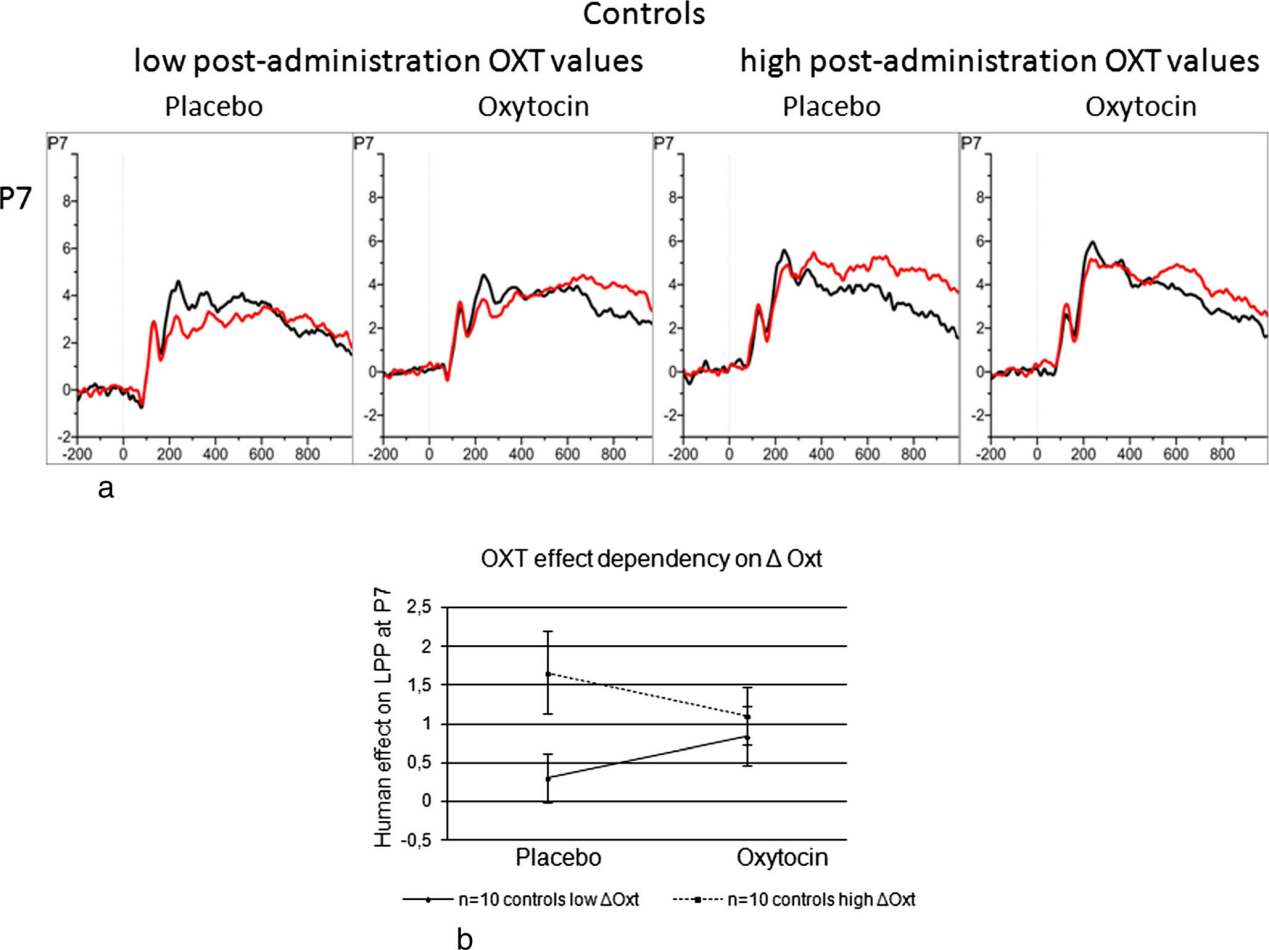
ERPs at P7 in response to pictures with and without humans of the control males having low (≤P33) and high (≥P66) post-administration blood plasma oxytocin values, respectively (**a**). Means and SEMs of the human effect illustrating the increase of the social orienting response in the OXT condition for only the males with low levels of post-administration blood plasma OXT. The graph also demonstrates the group differences in the placebo situation (**b**)

Summarizing the association of post-administration peripheral OXT with the OXT treatment effects, we found that individuals with low post-administration values showed an enhancement of their social orienting after OXT intake while those with high post-administration levels showed a decrease. Figure 7a, b illustrates the OXT administration-induced enhanced orienting to pictures with humans as is assessed by the LPP at P7 in only the low level (≤P33) post-administration OXT group, which differed significantly from the high level (≥P66) post-administration group [*F*_Treatment*Human*Valence(negative)*ΔOXTGroup_ (1,18) = 4.70; *p* = .04; *η*^*2*^ = .21]. The figures moreover show the significant difference between the groups in their placebo responses to pictures with humans.

## Discussion

The present study investigated whether OXT treatment effects on social orienting are moderated by blood plasma OXT levels. We first investigated whether there were any correlations between the plasma OXT levels and a variety of personality characteristics and behaviour problems as measured by questionnaires. No significant correlations were found, not even for the previously found OXT effect-moderating personality characteristics that were related to coping with social stress, anticipation of punishment and criticism or the drive for goal achievement (see Althaus et al. 2015). This corresponds with the findings by Jansen et al. (2006) who also did not find plasma OXT baseline levels to be correlated with any of their measures assessing autism- and anxiety-related behaviour in high-functioning adults with ASD or healthy controls.

Also in agreement with the study by Jansen et al. (2006), yet in contrast to what has been found in children with ASD (Modahl et al. 1998; Green et al 2001), is our finding that our group of ASD male adults had significantly *higher* baseline plasma OXT levels than our male adult control group. The difference with the findings in children was explained by Jansen and colleagues as being due to their sample consisting of intellectually high-functioning adults with ASD suggesting both developmental and intellectual factors playing a role in the increased OXT plasma levels. The suggestion of intellectual functioning being associated with OXT plasma levels in individuals with ASD is supported by the positive correlation we found between baseline plasma OXT and the level of intelligence in our high-functioning ASD group. This group, though not differing in mean IQ from our control group, showed significantly greater variance (Leven’s test: *F* = 8.14; *p* < .006) caused by several very high IQ scores (*n* = 7: IQ > 120). Considering that only the ASD participants with high baseline OXT levels showed an increase in their social orienting responses after OXT administration, and that baseline OXT levels were positively correlated with IQ, we investigated whether intelligence would be a moderator of the OXT administration effect but did not find so. Therefore, IQ seems to be functionally related to the baseline plasma OXT concentrations but not to the effect of exogenously administered OXT on social orienting.

The seemingly counterintuitive finding of male adults with ASD having higher plasma concentrations of OXT corroborates not only the finding by Jansen et al. (2006) but also those of several other studies. Higher plasma concentrations were also found in males with a major depression (MD) as compared to females with MD as well as to healthy male controls (Yuen et al. 2014). This gender dependency proves how important it is to take into account sex differences when studying the role of OXT in psychopathological conditions. Plasma concentrations were also shown to positively correlate with fear of romantic attachment in healthy (predominantly female) individuals (Marazziti et al. 2006) and with the severity of social anxiety and dissatisfaction with social relationships in male and female patients with a Generalized Social Anxiety Disorder (GSAD) (Hoge et al. 2008). Higher plasma OXT concentrations are hence not unique to male adults with ASD and are suggested to go along with social anxiety-related problems.

OXT release into the peripheral circulation, however, may depend on what happens shortly before and/or during blood collection. As both physical and psychological stress have been suggested to activate the hypothalamic-pituitary-adrenocortical (HPA) axis and as neuroendocrine stress responses have been characterized by, among others, OXT release from the pituitary gland into the peripheral circulation (e.g. Onaka 2004), one might argue that the venipunctures were more stressful to our ASD participants than to the control males leading to larger OXT releases into the bloodstream. There are several arguments which may weaken this hypothesis. In the studies by Modahl et al. (1998) and Green et al. (2001), children with ASD were involved showing *lower* plasma concentrations although they had undergone venipuncture as well. The present study included only participants with ASD who had indicated not to be afraid to undergo two venipunctures. Furthermore, no correlations were found between the decrease in OXT concentration and the decrease in the state anxiety measures obtained in the placebo situation, although the latter was shown to be significant in both groups (Althaus et al. 2015). If the punctures had been more stressful to the males with ASD leading to more peripheral OXT release, we would have expected a significant correlation between the decrease in STAI scores and plasma levels in the placebo situation, especially in the group of males with ASD.

Still more intriguing than the higher OXT baseline level found for the males with ASD is our finding that OXT administration effects on social orienting were moderated by peripheral OXT baseline concentrations. This moderation was opposite to what we had expected according to the literature. Assuming that the reported positive correlations of peripheral OXT levels with centrally controlled prosocial behaviour (e.g. Feldman et al. 2007; Strathearn et al. 2009) and negative correlations with psychopathological symptoms (e.g. Rubin et al. 2010; Skrundz et al. 2011) implicate that plasma OXT concentrations are reflective of intra-cerebral OXT concentrations and activity, we inferred a greater benefit from exogenously administered OXT in individuals with low peripheral levels of OXT. Yet, high levels of baseline plasma OXT were found to be associated with beneficial effects of nasally administered OXT on social orienting, while low levels were accompanied by a decrease in the orienting responses when OXT was administered. Figure 4 shows that the differences between individuals with high and low baseline plasma levels refer to the social orienting responses in only the OXT condition. Our findings therefore suggest that peripheral baseline OXT levels are predictive of the effects of extraneously administered OXT on centrally controlled social orienting but not of social orienting itself.

As the OXT treatment effects turned out to be beneficial in those who have higher baseline peripheral levels, we speculate that a high peripheral level might be indicative of an initially *lower* central baseline level which is likely to be enhanced by nasal administration of OXT. While the lacking correlations between plasma and CSF levels of OXT reported by Takagi et al. (1985), Winslow et al. (2003), Altemus et al. (2004), and Kagerbauer et al. (2013) have been supposed to reflect dissociated peripheral and central OXT release patterns, the positive correlations between absolute central and blood plasma *peak values* after nasal administration found in rats by Neumann et al. (2013) suggest the opposite. These findings, however, do not contribute to explain the direction in which we found the plasma baseline levels being associated with the central effects of nasally administered OXT. Might our findings on the associations of the treatment effects with the *post-administration* level shed some more light on the relationship between plasma OXT levels and the susceptibility to extraneously administered OXT? First, we found a significant and about tenfold increase of the plasma OXT values about 53 min (±SD = 4.92) after nasal administration. Adjusted for pre-administration values, these increases did not differ between our two groups of healthy male adults and male adults with ASD, neither in their absolute values, nor in their values relative to baseline. We further found that individuals with high post-administration OXT plasma levels showed decreased social orienting after OXT intake. This was assumed to be observed if higher peripheral post-administration levels reflect that less OXT had become centrally available or active due to leakage, less absorption or central saturation. Whereas intranasal applications of OXT have been shown to result in very large increases in circulating OXT i.e. to levels far above those that are needed for physiological effects, only small amounts may enter the brain while penetrating some specific brain regions yet not entering the CSF (Leng and Ludwig 2016; Neumann et al 2013). This may be due to leakage that has been suggested indeed to occur as a consequence of intranasal spray delivery. In studies on macaques, comparing nasal spray administration with OXT nebulizer delivery, a significant and similar elevation of CSF OXT levels was found for both delivery methods while plasma OXT was significantly enhanced only after intranasal administration (Dal Monte et al. 2014; Modi et al. 2014). The authors explained the significant elevation of plasma OXT by the possible formation of larger droplets from the spray, which would rest on the nasal mucosa and hence provide for vascular absorption as compared to the nebulizer forming a much finer mist and allowing more of the substance to be breathed in deeper towards the epithelium.

Yet, post-administration levels were not only related to the treatment effects (see Fig. 7a, b) but also to the social orienting responses in the placebo situation. The groups with low and high post-administration plasma levels differed in their ECR and LPP P7 responses to pictures with humans in especially the *placebo* situation (LPP at P7: *t* = 2.19; df = 18; *p* = .04; ECR: *t* = 1.8; df = 36; *p* = .08), which means that the groups differing in their post-administration OXT plasma levels also differ in their pre-administrative social orienting responses that are centrally controlled because they demand the evaluation of information as being relevant or not. This cannot be explained by differences in leakage of the administered OXT from the nasal mucosa into the bloodstream but might agree with the above proposed hypothesis of individual differences in central absorption being due to differences in central bioavailability. Individuals with lower post-administration plasma concentrations might have had an initially decreased *central* availability of OXT with the consequence that more of the OXT administered became centrally active while less was released into the peripheral bloodstream.

The study included only males with an IQ ≥ 80, with seven males of the ASD group having an IQ of even ≥120. This confines generalizability of our findings to a subgroup of only high-functioning males with ASD. As IQ was found to correlate with baseline OXT plasma concentrations in this group, OXT studies with low functioning individuals with ASD are warranted.

The number of participants was too small to enter multiple covariates into the design in order to investigate the potential interactive moderating of OXT effects by OXT baseline levels and the previously found questionnaire-assessed behaviour. Yet, for the behaviour that was previously found to moderate the effects of OXT on social orienting, no correlations were found with OXT baseline levels.

The findings on the OXT moderations cannot be interpreted as unambiguously specific to the two groups investigated, i.e. the findings on baseline OXT plasma concentration as moderating the treatment effect in only the males with ASD and the findings on post-treatment OXT concentrations as moderating the treatment effect in only the control group since group differences were not found to be significant.

Yet, from the findings of the present study, we can conclude that plasma OXT levels are associated with the effects of nasally administered OXT on social orienting as assessed by neurophysiological responses to socially relevant information. As our findings were rather unexpected, their interpretation is a tentative one; we propose that it is the central availability of OXT that determines how much of the nasally administered OXT will become absorbed and centrally active, and how much of it will become released into the bloodstream. Yet, still too little is known about the relationship between peripheral and central OXT concentrations on the one hand and peripheral concentrations and the central action of exogenously administered OXT on the other hand. This holds for healthy individuals as well as for individuals with a psychopathological condition and means that both more preclinical and clinical studies are warranted. Preclinical studies might, for example, compare OXT plasma and CSF or local brain OXT concentrations after both nasal administration and intracerebroventricular (ICV) infusion while at the same time investigating social behaviour. This has, to the best of our knowledge, not yet been done but would be an extension of, for example, the studies by Calcagnoli et al. (2015a, b) who compared the effects of ICV with intranasal OXT administration on aggressive and prosocial behaviour in rats but did not analyse plasma OXT concentrations, and by Neumann et al. (2013) who compared changes in central and peripheral OXT concentrations after nasal and intraperitoneal administration but did not relate these concentrations to central effects, i.e. to changes in social behaviour. Clinical studies on the effects of exogenous OXT administration (preferably by nasal spray) should more and more take into account peripheral OXT levels as a potential treatment moderator.
